# Research on the requirement characteristics and differentiated service design of home-based elderly care service in China using the candy model

**DOI:** 10.1038/s41598-025-21395-7

**Published:** 2025-11-03

**Authors:** Dianfeng Zhang, Tianya Xu, Yanlai Li, Shouyang Wang, Xiaoyu Dong, Mingze Yuan

**Affiliations:** 1 School of Management, Qufu Normal University, Rizhao, 276800 People’s Republic of China; 2 School of International Economics and International Relations, Liaoning University, Shenyang, 110036 People’s Republic of China; 3 Institute of Mathematical and Systematic Sciences, Chinese Academy of Sciences, Beijing, 100045 People’s Republic of China; 4 The School of Entrepreneurship and Management, Shanghai Technology University, Shanghai, 201210 People’s Republic of China

**Keywords:** Home-based elderly care, Interval Kano model, Grounded theory, Candy model, Importance satisfaction analysis, Ageing, Health care

## Abstract

In response to the escalating aging population in China, this study undertakes a comprehensive requirement analysis and differentiation exploration of home-based elderly care (HBEC) services. 46 HBEC service items across five dimensions are designed by adopting Grounded Theory, including life services, intelligent services, health services, security services and spiritual services. In terms of methodology, an optimized questionnaire based interval Kano model, namely Candy model is proposed and verified. The newly proposed model changes the way requirements are classified and identifies a new category of requirements: critical requirement. By conducting questionnaire surveys, 5 Critical (C), 7 Must-be (M), 21 One-dimensional (O), 6 Attractive (A), and 7 Indifferent (I) requirements are analyzed. The analysis result of importance shows that the importance order of the five kinds of requirements is: C > M > O > A > I. The correlation coefficients between importance and satisfaction/dissatisfaction are both bigger than 0.9, indicating a strong positive correlation between the importance and satisfaction sensitivity. Furthermore, the differentiation analysis is conducted considering factors such as income level, relationship with children and self-care ability. The differentiation of requirements often occurs in the upstream and downstream requirements within the dynamic change pattern “I → A → O(C) → M”. This study offers valuable results for addressing the challenges associated with population aging.

## Introduction

Meeting requirements is the fundamental motive behind user consumption; therefore, whether a product or service can satisfy those requirements directly determines the success or failure of its design^1^. The once-prevailing notion of merely providing “tools for sustaining life” has become obsolete, as it fails to address individuals’ increasingly diversified and personalized requirements^2^. With the ongoing transformation^3^, service design must undergo a paradigmatic shift from a supply-centric to a demand-oriented approach^4^, one that intensively centers on users’ authentic needs and situated experiences. At the same time, service design has increasingly turned its attention to consider stakeholders’ influences^5^, since understanding their requirements helps service providers better coordinate relationships among stakeholders and more effectively achieve their objectives^6^. These transitions are particularly crucial when confronting China’s distinctive aging challenge, which is characterized by a deeply rooted culture of filial piety, an enormous elderly population, an accelerated pace of aging, and the “getting old before getting rich” phenomenon^7^. As the aging issue continues to intensify, there is an urgent imperative to conduct elderly-care service design research grounded in the demands of multiple stakeholders. Resolving China’s aging dilemma is not only vital for domestic welfare but also poised to offer valuable insights for other nations facing similar pressures.

The seventh national population census conducted in 2021 revealed that China has 264.02 million individuals aged 60 and above, comprising 18.70% of the country’s total population, and the proportion of the elderly population has increased by 5.44 percentage points compared to 2010. This trend is expected to continue rising rapidly because of the three distinct baby boom periods, including 1950–1957, 1962–1971 and 1981–1992^8^. The increasing number of people from the three baby boom periods and the increasing lifespan are accelerating the aging process in China. The issue of elderly care is further complicated by the effects of shifts in family structure and relationship^9^, socio-cultural transformations^10^, economic development characteristics^11^, and rapid urbanization, all of which are driven by the declining fertility rate^12^. The “4-2-1” and “4-2-2” family structures have become common in China, significantly increasing the burden on the younger generation^13^. The traditional elderly care mode, which solely relies on children to care for elderly family members, can no longer fully accommodate the current elderly care landscape^14^, necessitating the refinement of new elderly care modes.

Home based elderly care (HBEC) emphasizes that the elderly continues to receive care in their own homes. Unlike family-centered care, the service providers for HBEC are not confined to family members but can also encompass social organizations and individuals. Compared to institutional care, HBEC enables the elderly to enjoy greater autonomy and comfort^15^, without being affected by factors such as unstable service levels, high costs, and disconnection from their previous lives^16^. HBEC not only fulfills the psychological needs of the elderly but also alleviates the pressure on younger generations and governments, thereby balancing the contradiction^17^. Kaye et al. pointed out that compared to other elderly care models, HBEC alleviates the pressure on family caregivers and eases the financial burden on the government^18^. A larger contingent of scholars discusses potential solutions for old-age care by adopting HBEC. Laporte et al. pointed that HBEC enables individuals not only to reside in a comfortable familial setting, but also better and more flexibly meet the needs of elderly care^19^. Shao et al. highlighted that fostering the harmonious development of social organizations involved in home care services could offer viable answers to the escalating requirement for HBEC services^20^.

Consequently, the HBEC has garnered substantial attention from scholars. Researchers have focused on the quality of life of HBEC services^21^, analyzed the types of service requirements of the elderly^22^, studied their influencing factors^23^, and actively promoted the deep integration of home care and intelligent technology^24^. These efforts have provided valuable insights and profound enlightenment for the future trajectory of HBEC development. However, current scholarly research has some deficiencies. Previous studies have yet to delve into whether the residences of the elderly are suitable for aging life, how to ensure the safety of property within these residences, and how to manage the relationships among the elderly, their children, service agencies, and service personnel. Furthermore, there remains a lack of diversified and multi-tiered studies on the service requirements of different segments of the older population. Meanwhile, a multitude of technical means enrich the lives of the elderly across medical health, social activities, spiritual comfort, and other aspects, catering to their diverse requirements^25^. Therefore, it is not only imperative to discuss the requirement analysis and service design research of HBEC services in the context of digital intelligence, but such discussions also hold far-reaching significance^26^.

**Fig. 1:**
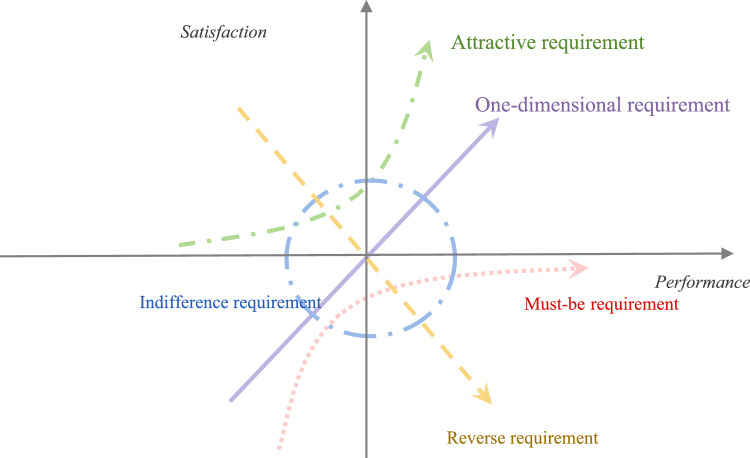
The Kano model.

In terms of methodological approaches, previous studies have employed the Logistic model^27^, decision tree model, and, notably, the Kano model. Compared to other methodologies, the Kano model yields more diverse and specific outcomes, which can be more directly translated into service design and optimization efforts^28^. The Kano model is commonly employed to identify factors influencing customer satisfaction (CS), enabling systematic identification of satisfaction improvement levers^29^. The underlying principle of the Kano model posits that satisfaction and dissatisfaction with attribute performance vary non-linearly, and the relationship between these two sentiments is not a simple symmetric one, but rather encompasses a multitude of complex interactions (Fig. 1).

However, the application of the traditional Kano model faces challenges in the context of elderly care in China. The characteristics of certain groups’ requirements may be submerged within the larger group, and the differences within the focused requirements cannot be well represented^30^. Therefore, there is a need for more nuanced models that can better capture inter-group differences. Building upon this foundation, this paper proposes the Interval Kano model, also referred to as the Candy model, which is an advancement of the traditional questionnaire-based Kano model. By utilizing a ratio algorithm, this model mitigates the masking effect of absolute values on differences and further refines the hierarchy of needs through the incorporation of quadrants. This enhancement aims to improve the stability, diversity, and granularity of the results.

Building upon the aforementioned discussion, this study undertakes an in-depth exploration of specific HBEC service items, taking into account the uniqueness of the setting and the application of intelligent technology. To summarize, this study offers three primary research contributions. Firstly, this study innovates grounded theory (GT)^31^ application by incorporating heterogeneous online data beyond traditional interviews, enabling systematic analysis of HBEC requirements from older adults and their children. The resulting service items are then subjected to an effectiveness test that combines importance weights from a questionnaire pre-survey with Kano attribute classifications, and items are subsequently added or removed to ensure that the proposed service portfolio is both valid and actionable. Secondly, building upon the traditional Kano model, this study introduces the Candy model, specifically designed for analyzing the characteristics of HBEC. This model provides a clearer portrayal of inter-group requirements disparities. Thirdly, leveraging systematic service content design and methodological innovation, this study delves into the differentiated requirements among elderly groups based on factors such as income level, health conditions, and relationships with their children. This comprehensive study provides an insightful portrait of the content and mechanism of the requirements for HBEC services in China.

## Related works

### Research status of elderly care services

Previous studies have encompassed numerous facets of elderly life, with medical health being the foremost concern due to a scholarly consensus regarding the frail physical condition of the elderly^32^. In terms of other dimensions, scholars have predominantly focused on the requirements for material life, spiritual life, or entertainment, while only a minority has examined the requirements pertaining to legal requirements, security requirements, and other pertinent requirements (Table 1).

**Table 1:** Research on elderly care service requirements.

References	Elderly requirements	Research conclusion
Chen et al.^33^	Feeding, dressing, bathing, grooming, toilet use, chair/bed transfer, walking and using stairs, etc	The unmet needs with disabilities were serious, with a high proportion of unmet needs to get out of the bedroom and for activities involving mental aspects
Li et al.^34^	Health care	This study revealed the difference between urban and rural health care of the elderly and its influencing factors
Li et al.^35^	Medical care services, medical care, rehabilitation services, psychological support and recreational activities	The elderly population has the greatest need for medical care services, followed by professional rehabilitation assistance counseling, guidance, and training
Zhu^36^	Security and convenience needs, social and medical needs, smart home technology	People were most concerned about health needs, smart devices were widely used, but the degree of aging design was low
Wang et al.^37^	Smart aged care needs: medical care, spiritual comfort, recreation, emergency ambulance and life care	The elderly had a low level of understanding of smart elderly care, but had high requirement, which was affected by individual, family, health status, and others
Chen et al.^38^	Mental health services	Older people living with family members were mainly manifested as the need for social interaction, belonging and self actualization. Older people living alone in special groups mainly want support to deal with loneliness

Within the domain of HBEC, extant scholarship is marked by two conspicuous gaps. First, empirical attention to the home-care scenario remains scant; investigations into the heterogeneity and diversity of older adults’ needs are neither sufficiently representative nor comprehensively scoped. Second, extant syntheses of these needs are underdeveloped. Research on smart-home technologies has disproportionately emphasized technical innovation and policy endorsement, while neglecting their systematic integration into care workflows. Moreover, requirement analyses have been circumscribed to the elderly cohort per se, thereby overlooking the strategic interactions of familial caregivers—most notably adult children—whose preferences and constraints co-determine the implementation of care arrangements. Although the requirements for intelligence has garnered increasing scholarly attention^39^, no research has yet fully analyzed the characteristics of intelligent product requirements from the perspectives of scene specificity^40^, elderly characteristics, and the interplay among stakeholders such as children and other involved parties. On this basis, in terms of service content design, the present study intends to design service content that takes into account the uniqueness of the scene, group differences, and the harmonious integration of intelligence across multiple stakeholders. This endeavor will delineate a more comprehensive, systematic, and scientifically rigorous requirement map for the elderly population.

In terms of the research methodologies employed for requirement analysis, satisfaction analysis, and service design in the context of HBEC, scholars primarily utilize decision trees^41^, dimensional analysis^42^, regression models^43^, and the Kano model. The Kano model, proposed by Noriaki Kano, examines the nonlinear relationship between attribute performance and CS, emphasizing that satisfaction and dissatisfaction are asymmetric^44^. The comparative strengths and limitations of these various approaches are outlined in Table 2. Kano model provides a satisfaction index that elucidates the magnitude and manner in which different attributes influence satisfaction. This explanatory capability regarding how various attributes affect overall CS is a unique feature not possessed by regression models and other methodologies, especially in terms of CS oriented design^45^.

**Table 2:** Comparison of common methods for elderly care service requirement analysis.

Method	Method characteristics	Strengths and weaknesses
Classification tree method	The whole variable or explanatory variable is decomposed hierarchically according to the classification characteristics and the progressive relation	The advantage is that it can fully mine user requirements and facilitate classification and interpretation of progressive relations. The main disadvantages are limited by the ability of researchers and cannot reflect the nonlinear characteristics of specific needs
Logistic regression	Taking overall CS or specific requirement selection behavior as the dependent variable, the influencing factors are analyzed	The advantages are wide application, mature, intuitive logic and simple. The main disadvantage is that the CS or requirement as a whole analysis cannot reflect the nonlinear characteristics of specific needs and diversified differences
Dimensional analysis	By setting two or more categorical variables as the basis for quadrant division, the groups are classified and analyzed	The advantages are wide application, simple operation, suitable for qualitative research and flexible application forms. The disadvantages of the results are poor applicability, strong subjectivity, and cannot reflect nonlinear differences
Kano model	The nonlinear characteristics of multiple different attributes can be identified by measuring the CS difference of the same attribute in two opposite cases	The advantages are that it can reflect the characteristics of nonlinear requirements, facilitate classification analysis and importance ranking, quantitative calculation and simple calculation. The main disadvantage is the accuracy of questionnaire calculation among different cases

### Research status of Kano model

The Kano model categorizes customer requirements into must-be (M), one-dimensional (O), attractive (A), indifference (I), and reverse (R) types, with the first three being of paramount importance. Notably, the impact of O and A diminishes by approximately 30% if M requirements are not met (Tontini et al.)^46^. According to the hierarchy of requirement types, the priority of satisfaction should adhere to the sequence: M > O > A > I^47^. In distinguishing service attributes, researchers typically utilize the modified Kano survey results analysis table (Table 3).

**Table 3:** Comparison table of Kano model evaluation results.

Project		Reverse question				
		I don’t like it	Can bear	It doesn’t matter	Ought to be	Be fond of
Forward question	I don’t like it	Q	R	R	R	R
	Can bear	M	I	I	I	R
	It doesn’t matter	M	I	I	I	R
	Ought to be	M	I	I	I	R
	Be fond of	O	A	A	A	Q

A substantial body of literature has applied the Kano model to the design of ageing-related services, including smart-home solutions for older adults^48^, mobile reading for older adults^49^, public sports service for older adults^50^, intelligent medication administration system for older adults^51^, healthy diet App for older adults^52^, and healthcare robots at home for older adults^53^. Simultaneously, the model has transcended both research subjects and sector boundaries, informing service designs for children^54^, urban Chinese youth^55^, persons with disabilities^56^, breast cancer survivors^57^, tourists^58^, real estate consumer^59^, and daily commuters^60^. This breadth of application offers a robust reference for the present study.

The traditional Kano model exhibits certain limitations, prompting scholars to enhance it through various methodologies^61^. Although the traditional Kano model has been refined through a variety of methodological extensions, important limitations persist. Li integrated AHP, QFD and IFS to address new-product development decisions, yet the approach remains vulnerable to subjective judgment^62^. Cai et al. constructed a fuzzy Kano model by assigning different language values to different options, thereby enhancing the discrimination and data representativeness, but the fuzzy functions and their thresholds still lack an empirical basis^63^. Yang et al. proposed a BERT-TCBAD-Kano hybrid to analyse new-energy vehicles, mitigating the bias induced by imbalanced online reviews; however, the model draws on a single data source and relies on the mean value alone for attribute classification, undermining its robustness^64^. Collectively, these studies have only partially resolved the traditional model’s tendency to obscure inter-group heterogeneity, and none has addressed the extreme distribution of needs that characterises China HBEC context—most notably the clustering of one-dimensional requirements. An HBEC-tailored Kano model is therefore urgently required.

Influenced by traditional concepts, some families adhere to more conventional practices, where children bear the primary responsibility for elderly care^65^. During the survey, such respondents tended to treat the listed services as supplementary rather than essential, judging satisfaction and importance on the assumption that they themselves already cared for the older adult or that the older adult remained fully self-reliant. Consequently, most items elicited neither pronounced satisfaction nor dissatisfaction; only a handful of options revealed any discernible preference. Consequently, the use of traditional methods often yields a substantial number of indifferent requirements^66^. Simultaneously, due to a preoccupation with their immediate interests, some respondents in the Kano questionnaire exhibit a heightened concern for part of elderly care services. Consequently, when completing the questionnaire, they often tend to select the two extreme values of “I don’t like it” and “Be fond of” for a certain service item. Therefore, the survey results would present an abundance of one-dimensional requirements^67^. This has led to the results of the survey being distributed near the midline of the first and third quadrants, a pattern that has been observed in many previous studies^30^. Under such circumstances, the heterogeneous signals of strong demand for specific services are submerged within homogenised data, making it impossible to discern their respective contributions to enhancing satisfaction or eliminating dissatisfaction^68^.

To address this issue, this study draws inspiration from the innovation of the online review Kano model and employs a ratio algorithm to effectively mitigate the interference caused by partially homogeneous questionnaires^69^. This approach enables a precise extraction of volatility information within the questionnaires. However, it becomes challenging to demonstrating the intensity disparity of internal attributes between one-dimensional and other requirement categories. To address this, the present study introduces an interval Kano model grounded in questionnaire surveys. We have named the interval Kano model the Candy Model. Its key distinctions from other models are summarized in Table 4.

**Table 4:** Comparison between the Candy model and existing models.

Evaluation dimension	Kano model	BW model	Candy model
Valid requirement category	4	4	5
Adaptation type	High-quality questionnaire	Minimal disparity across requirement categories	Homogeneous data with pronounced outliers
Partitioning dimension	Frequency	Mean value	Interval value

## Methods

**Fig. 2:**
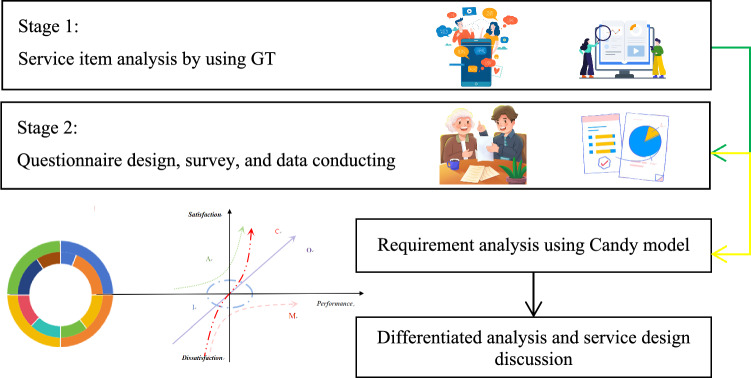
Study process.

This study first employs GT analysis to extract multi-source heterogeneous data from documents and then summarizes and generalizes specific service items. Subsequently, a Kano questionnaire survey, which includes the importance survey, is conducted to investigate the requirements. Next, an interval Kano model, namely the Candy model, is proposed to analyze the questionnaire to obtain information on the characteristics of requirement categories and importance. Finally, based on the sample statistical information, a differentiated requirements feature analysis is carried out to explore the satisfaction and importance characteristics of different groups towards the service items (Fig. 2). The study has been performed in accordance with the Declaration of Helsinki. We confirm that informed consent was obtained from all participants and/or their legal guardians. We also confirm that the experimental protocol/s was/were approved by Biomedical Ethics Committee, Qufu Normal University.

### Design of service items

To address the limitation of systematic and comprehensive analysis of service projects in prior research, this paper innovatively conducts a field investigation drawing upon forum data, domestic and international literature, and domestic realities to derive the service items and categories of HBEC services. This paper employs GT to examine these textual data and validates the plausibility of the resulting service requirements through an importance survey. Ultimately, 46 types of service requirements are identified in this study. By transcending the constraints of single-source data, this process enhances both the comprehensiveness and the validity of service elicitation, thereby filling the extant research gap in data integration during the service-identification phase. Based on an in-depth understanding and comprehensive consideration of the unique attributes of these service items, they are logically categorized into five groups: life services, intelligent services, health services, security services, and spiritual services (Table 5).

**Table 5:** Content framework of HBEC service.

Category	Service item	Code
Living needs	Meal delivery service	A1
	Indoor cleaning service	A2
	Item storage solution	A3
	Maintenance service	A4
	Heavy lifting service	A5
	Indoor anti-slip and anti-fall service	A6
	Pet walking service	A7
	Daily necessities purchasing service	A8
	Sewing and repair service	A9
	Electronic product use training	A10
	Attire matching guidance service	A11
Intelligent needs	Wearable emergency call device	B1
	Intelligent reminders for going out	B2
	Intelligent chat terminal system	B3
	Household robot	B4
	Smart security door lock	B5
	Surveillance equipment system	B6
Health needs	Diet and health training program	C1
	High-end nutritious meals	C2
	Regular physical examination service	C3
	Medication purchasing service	C4
	Home haircut service	C5
	Assistance with hair washing or bathing	C6
	Rehabilitation training and nursing care	C7
	Wheelchair-assisted walks service	C8
	Online medical consultation service	C9
	Tuina massage service	C10
	Preparation of chinese medicine	C11
Security needs	Legal consultation service	D1
	Anti-fraud publicity campaign	D2
	Service personnel complaints handling	D3
	Emergency call response system	D4
	Service personnel reliability audit	D5
	3-party indoor surveillance system	D6
	Service process recording	D7
	Indoor property registration service	D8
	Regular trauma examination service	D9
	Refusal to accept inheritance service	D10
Spiritual needs	Chat and social activities	E1
	Psychological counseling service	E2
	Musical activities	E3
	Hobby-based activities	E4
	Board and card games	E5
	Senior social events	E6
	Square dancing	E7
	Traveling	E8

The design of the service content is grounded in a diverse array of references, while integrating considerations such as the distinct characteristics of the elderly population, the unique aspects of the home care setting, the multifaceted needs of stakeholders, and advancements in smart technology. Furthermore, acknowledging that diverse groups may harbor varying needs, the questionnaire design in this study adopts a stratified approach to the same service content. It proposes differentiated meal delivery services tailored to different income brackets and offers optional, differentiated services for individuals with varying physical conditions. In terms of service content design, this study endeavors to ensure the comprehensiveness of the service offerings, while simultaneously reflecting the disparities stemming from different roles, income levels, relationships with children, geographical distance from children, self-care capabilities, and other pertinent factors.

### Candy model

There are five options in the options Settings, which are “I don’t like it”, “Can bear”, “It doesn’t matter”, “Ought to be”, and “Be fond of”. Let $M_{i}$ indicate these options, where $i \in I$, and $I \in \left\{ {1,2,3,4,5} \right\}$, then $M_{i} = i$ can be set in the forward question and $M_{i} = 6 - i$ can be set in the reverse question. Order $N_{j}$ indicates the $j$ attributes or service items, where $j \in J$, and $J \in \left\{ {1,2,3,...,i...,n} \right\}$. Based on this, we can let $X_{ij}$ represent the option values $M_{i}$ and $X_{ij} \in I$ of the respondent’s choice among attributes $N_{j}$. Denote $Q_{k}$ as the respondent, $k \in K$, and $K \in \left\{ {1,2,3,...,k,...,q} \right\}$. Then respondent $Q_{k}$ can select values $X_{kij}^{pos}$ in the positive question of an attribute, and values $X_{kij}^{neg}$ in the negative one.

Let $\alpha_{j}^{pos}$ represent the positive satisfaction scores for attribute $N_{j}$, while $\alpha_{j}^{neg}$ denotes the negative satisfaction scores for the same attribute. By definition, the two sets of values can be computed using the following formula:

1 $$\alpha_{j}^{pos} = \sum\limits_{k = 1}^{q} {X_{kij}^{pos} },$$

2 $$\alpha_{j}^{neg} = \sum\limits_{k = 1}^{q} {X_{kij}^{neg} }.$$

Let $\beta_{j}^{pos}$ indicates satisfaction and let $\beta_{j}^{neg}$ indicates dissatisfaction, then the former absolute value can be converted to relative value according to the following function:

3 $$\beta_{j}^{pos} = {{\alpha_{j}^{pos} } \mathord{\left/ {\vphantom {{\alpha_{j}^{pos} } {\sum\limits_{j = 1}^{n} {\alpha_{j}^{pos} } }}} \right. \kern-0pt} {\sum\limits_{j = 1}^{n} {\alpha_{j}^{pos} } }},$$

4 $$\beta_{j}^{neg} = {{\alpha_{j}^{neg} } \mathord{\left/ {\vphantom {{\alpha_{j}^{neg} } {\sum\limits_{j = 1}^{n} {\alpha_{j}^{neg} } }}} \right. \kern-0pt} {\sum\limits_{j = 1}^{n} {\alpha_{j}^{neg} } }}.$$

**Fig. 3:**
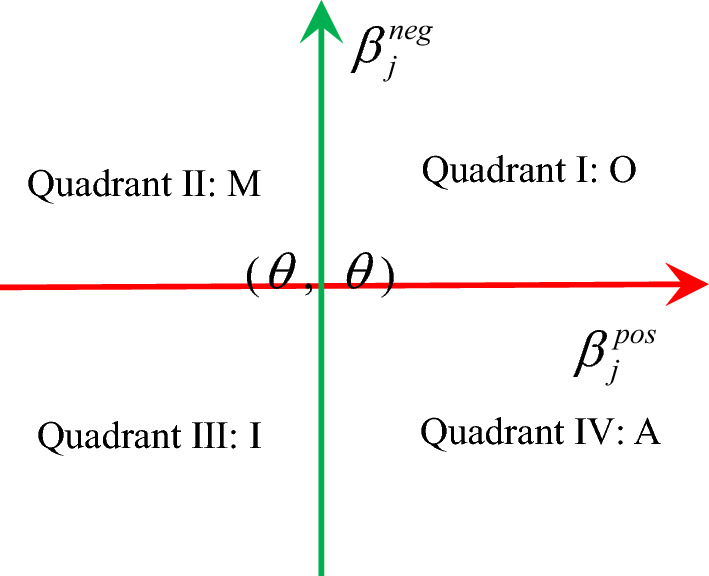
Four-quadrant model.

Since this data is structured questionnaire data, the respondents are all selected in the given range $\left\{ {1,2,3,4,5} \right\}$, so the floating ranges $\beta_{j}^{pos}$ and $\beta_{j}^{neg}$ are symmetric with each other, so only a single ratio conversion is required. According to the calculation rules, $\beta_{j}^{pos}$ and $\beta_{j}^{neg}$ have been normalized, and they have the same mean value $\theta$, where $\theta = {1 \mathord{\left/ {\vphantom {1 n}} \right. \kern-0pt} n}$. Therefore, this value can be used as the center points($\theta$, $\theta$) for constructing the four-quadrant model (Fig. 3).

The four-quadrant model is also employed in the BW model. The BW model categorizes requirements by computing the Better coefficient ($SI$) and the Worse coefficient ($DSI$). The fundamental partitioning concept aligns closely with Fig. 3, and a comparison of the results will be presented later in this paper. Specifically, the $SI$ and $DSI$ are calculated as the ratio of the number of specific attributes within a given attribute to the number of other attributes.

5 $$SI = {{\left( {A + O} \right)} \mathord{\left/ {\vphantom {{\left( {A + O} \right)} {\left( {A + O + M + I} \right)}}} \right. \kern-0pt} {\left( {A + O + M + I} \right)}},$$

6 $$DSI = - 1 \times {{\left( {O + M} \right)} \mathord{\left/ {\vphantom {{\left( {O + M} \right)} {\left( {A + O + M + I} \right)}}} \right. \kern-0pt} {\left( {A + O + M + I} \right)}}.$$

**Fig. 4:**
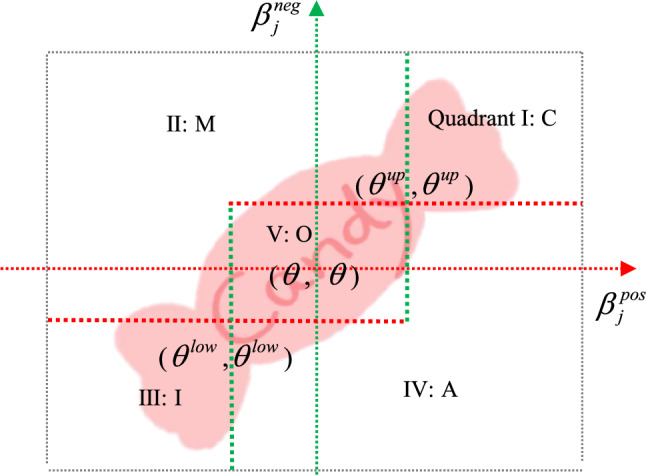
Five-quadrant diagram of the Candy model.

Comparing the studies of the two methods, it can be seen that the method of converting absolute value into relative value in this study is obviously different from that of BW model. The BW method results in the ratio of the number of respondents who make different choices, while the ratio method calculates relative satisfaction in the satisfaction and dissatisfaction domains respectively. Based on this, the determination of one-dimensional in the results obtained by the ratio method should be reconsidered. Because the connotation of the one-dimensional requirement is that satisfaction and dissatisfaction are similar, this means that this category should be located around the central point ($\theta$, $\theta$). Based on this, the interval ratio model is proposed. According to the shape of this model, this study named it the “Candy Model” (Fig. 4).

**Fig. 5:**
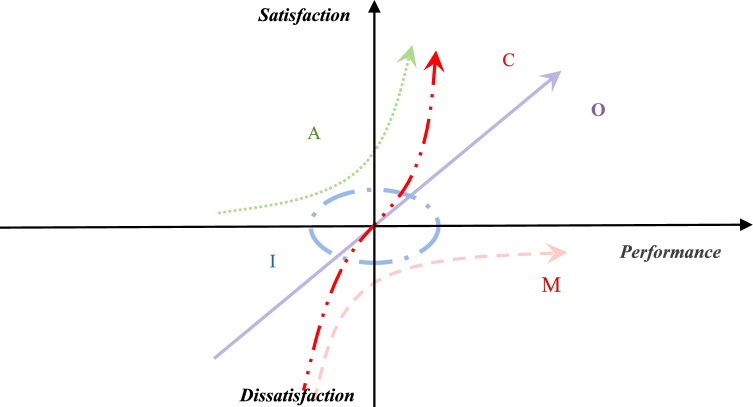
Five requirement features of the interval Kano model.

Unlike the ratio algorithm, which uses a central point to divide the quadrants, the interval Kano model replaces the clear boundary with an interval value of $\left[ {\theta^{low} ,\theta^{up} } \right]$. Based on this, the graphs of these five categories in the interval of satisfaction and performance are shown in Fig. 5.

In order to clarify the range of $\theta^{low}$ and $\theta^{up}$ values, ensure the symmetry of the two values, and incorporate the number of attributes $n$ into the values of the interval, we proposed the calculation formulas. The formulas for calculating the $\left[ {\theta^{low} ,\theta^{up} } \right]$ are given as follows:

7 $$\theta^{low} = \left( {1/n} \right) - v\phi,$$

8 $$\theta^{up} = \left( {1/n} \right) + v\phi,$$

9 $$\phi = \max \left\{ {\beta^{pos} ,\beta^{neg} } \right\} - \min \left\{ {\beta^{pos} ,\beta^{neg} } \right\},$$

where $\phi$ is the floating range of satisfaction and dissatisfaction. Refer to the study of Chen et al.^70^, $v$ is the floating ratio, and its value is about 1/6.

Based on the fluctuating boundary values of the intervals, the Candy model can determine the specific characteristics of the demand categories for service items using the following formula:

10 $$\left\{ {\begin{array}{*{20}l} {N_{j} \in O,\begin{array}{*{20}c} {} & {if} & {\begin{array}{*{20}c} {\theta^{low} < \beta_{j}^{pos} < \theta^{up} ,} & {\begin{array}{*{20}c} {and} & {\theta^{low} < \beta_{j}^{neg} < \theta^{up} } \\ \end{array} } \\ \end{array} } \\ \end{array} } \hfill \\ {N_{j} \in A,\begin{array}{*{20}c} {} & {if} & {\begin{array}{*{20}l} {\beta_{j}^{pos} > \theta^{low} ,} \hfill & {\beta_{j}^{neg} < \theta^{up} ,\begin{array}{*{20}c} {and} & {N_{j} } \\ \end{array} \notin O} \hfill \\ \end{array} } \\ \end{array} } \hfill \\ \begin{gathered} N_{j} \in M,\begin{array}{*{20}c} {} & {if} & {\begin{array}{*{20}c} {\beta_{j}^{neg} > \theta^{low} ,} & {\beta_{j}^{pos} < \theta^{up} ,\begin{array}{*{20}c} {and} & {N_{j} } \\ \end{array} \notin O} \\ \end{array} } \\ \end{array} \hfill \\ N_{j} \in C,\begin{array}{*{20}c} {} & {if} & {\beta_{j}^{pos} > \theta^{up} ,\beta_{j}^{neg} > \theta^{up} } \\ \end{array} \hfill \\ \end{gathered} \hfill \\ {N_{j} \in {\text{I,}}\begin{array}{*{20}c} {} & {\begin{array}{*{20}c} {if} & {\beta_{j}^{pos} < \theta^{low} ,\beta_{j}^{neg} < \theta^{low} } \\ \end{array} } \\ \end{array} } \hfill \\ \end{array} } \right.$$

The modified model can effectively limit the one-dimensional requirements within a reasonable range, and the model shows enough flexibility in the classification of different attributes, and can identify the requirement category more accurately, thus improving the stability and diversity of results.

## Results

### Questionnaire survey

This survey utilized a standardized Kano model questionnaire which also incorporates the importance ratings of service items. The questionnaire was primarily distributed to family members and students enrolled in QFN university, with the surveyed population primarily originating from ShanDong province and its adjacent regions in China (Table 6). The survey was conducted between Aug. 2024 and Oct. 2024. The survey instrument was designed and administered through the “Questionnaire Star” platform. A total of 483 individuals participated, and 425 ones completed the survey. Following preprocessing, 370 valid questionnaires were obtained, yielding an overall effective response rate of 87.06%.

**Table 6:** Questionnaire survey sample characteristics.

Category	Options	Quantity	Rate
Sex	Male	86	23.24%
	Female	284	76.76%
Residential area	First and second-tier cities	80	21.62%
	Ordinary city	98	26.49%
	County	92	24.86%
	Village	100	27.03%
Fill in the role	Elderly perspective	187	50.54%
	Child perspective	183	49.46%

SPSS 22.0 software was employed to assess the reliability and validity of the questionnaire data. The results of the reliability test revealed that the overall Cronbach’s α coefficient of the questionnaire was 0.937, indicating excellent reliability. Specifically, the Cronbach’s α for the forward questions was 0.886, and for the reverse questions, it was 0.889; both values exceeded the threshold of 0.8, confirming the questionnaire’s strong internal consistency. In terms of validity (Table 7), the overall KMO measure of the questionnaire was 0.921, suggesting high sampling adequacy. The KMO values for the forward and reverse questions were 0.910 and 0.907, respectively, both surpassing the 0.90 benchmark, which underscores the strong factorial validity of the data. Furthermore, the significance level of the Bartlett’s Test was less than 0.001, indicating that the correlation matrix was suitable for factor analysis and confirming the good quality of the data, thereby rendering it appropriate for subsequent analytical procedures.

**Table 7:** Reliability and validity analysis.

	Cronbach’s *α*	KMO	Degrees of freedom of Bartlett’s test
Forward question	0.886	0.910	0.000
Reverse question	0.889	0.907	0.000
Overall questionnaire	0.937	0.921	0.000

### Descriptive statistical analysis

When analyzing the basic information of the survey respondents, it was found that most elderly people’s educational levels were concentrated at the primary or junior high school level, and their educational level is generally low, which is consistent with the findings of previous scholars^71^. In terms of family background, asset status, physical health and other dimensions, the specific distribution of respondents is shown in Table 8.

**Table 8:** Descriptive statistics of survey samples.

Variable name	Percent	Variable name	Percent
*The spouse of the elderly*		*The balance sheet of the elderly*	
Widowed, unmarried, separated, alone	22.46%	Assets or cash are insufficient to cover debts	3.74%
Poor relationship or distrust	2.67%	A small amount of assets and cash	36.90%
General relationship	18.18%	Some assets or cash are relatively easy	56.68%
On good terms	56.68%	To be rich with a lot of assets or cash	2.67%
*The economic income level of the elderly*		*Geographical distance between elderly and children*	
2000 yuan and below	37.97%	Live together or in close proximity	48.38%
2001–4000 yuan	35.29%	Living in the same city	25.14%
4001–6000 yuan	9.63%	Travel within 3–5 h	17.84%
More than 6001 yuan	17.11%	The trip takes about 6–12 h	5.14%
–	–	The trip takes more than 12 h	3.51%
*Self-care ability oof the elderly*		*Relationship between elderly and children*	
Total loss of self-care	0.45%	Very distant	0.00%
Heavily dependent	0.76%	More distant	0.00%
Heavily depend on	1.21%	Occasional contact	1.08%
Moderate dependence	3.63%	General relation	3.78%
Mild dependence	9.37%	closer	11.35%
Basic self-care	20.69%	Very close	25.95%
Complete self-care	63.90%	Closely dependent	57.84%

### Requirement category analysis

**Fig. 6:**
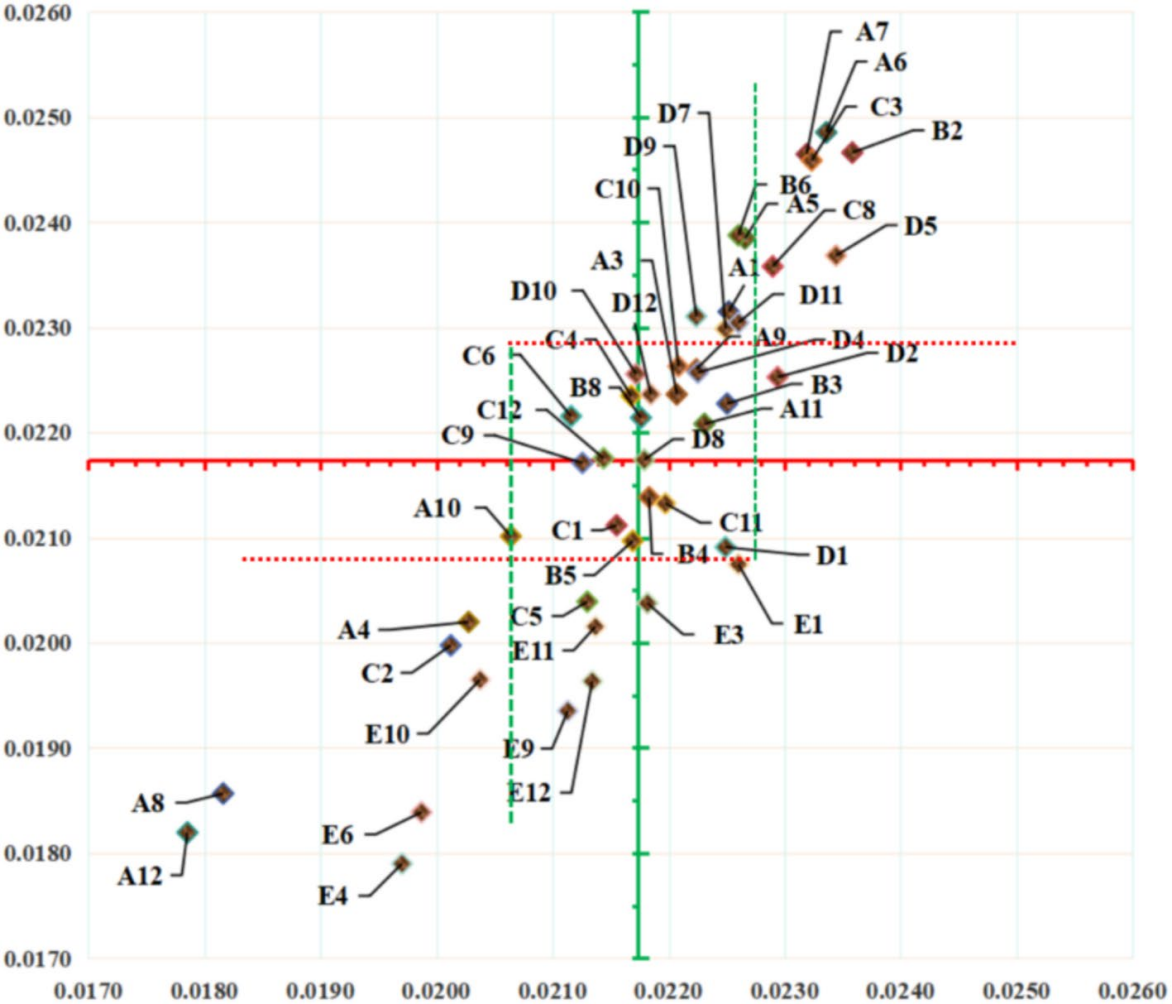
Analysis results of ratio algorithm and Candy model.

Utilizing formulas (1)–(4), the absolute data collected through the questionnaire can be transformed into relative data. According to the established calculation rule, the mean value for both satisfaction and dissatisfaction is equivalent, denoted as $\theta = 0.02174$. Utilizing formulas (7)–(9), the value of $\phi$ can be determined. Furthermore, by referencing the analytical findings of other scholars in comparable studies, the value in this particular research is established as $v = 1/6$. On this basis, the range of $\theta$ can be derived as $\left[ {\theta^{low} ,\theta^{up} } \right] = \left[ {0.02057,0.02291} \right]$. Utilizing the interval values associated with $\theta$, a five-quadrant model can be constructed (Fig. 6).

As illustrated in Fig. 6, the requirement types of service items are predominantly concentrated within the first, third and fifth quadrants, with a notable clustering around the central point. Services in the first, second, forth, and fifth quadrants are intrinsically tied to the elderly’s fundamental well-being, while any improvement or deterioration in these services can significantly impact their satisfaction attitudes. In terms of satisfaction degree variation, some service contents are situated close to the central point, whereas others are situated farther away. This indicates a substantial difference among the service contents distributed within the same quadrant, suggesting varying degrees of importance and impact on satisfaction.

Subsequently, based on the results obtained through the traditional method, this study calculates the requirement category for service items using the BW method, guided by formulas (5) and (6). As presented in Table 9, the outcomes reveal that both the traditional method and the BW method encompass a substantial number of indifferent and one-dimensional requirements^66^. The results obtained through both the traditional method and the BW method rely heavily on absolute values, which inherently lack the capability to discern differential data from similar information. Consequently, these results may diverge from the actual requirements of the target user group. Users who regard services as irrelevant or inconsequential are unlikely to become genuine adopters of HBEC. The Candy model compensates for this limitation by converting absolute scores into relative indices, thereby enabling more effective extraction of actionable insights.

**Table 9:** Requirement analysis results for service items using different methods.

Service content	Importance	$\beta_{j}^{pos}$	$\beta_{j}^{neg}$	Ratio model	Candy model	Normal method	BW method
A1	4.2838	0.0225	0.0231	O	M	O	O
A2	4.1811	0.0221	0.0224	O	O	I	O
A3	3.6973	0.0203	0.0202	I	I	I	I
A4	4.3919	0.0227	0.0238	O	M	O	O
A5	4.5946	0.0234	0.0249	O	C	O	O
A6	4.6541	0.0232	0.0246	O	C	O	O
A7	3.2730	0.0182	0.0186	I	I	I	I
A8	4.2324	0.0222	0.0226	O	O	I	O
A9	3.7946	0.0206	0.0210	I	O	I	I
A10	4.2162	0.0223	0.0221	O	O	I	A
A11	3.0027	0.0179	0.0182	I	I	I	I
B1	4.6405	0.0236	0.0247	O	C	O	O
B2	4.2135	0.0225	0.0223	O	O	I	O
B3	3.8297	0.0218	0.0214	A	O	I	I
B4	3.5703	0.0217	0.0210	I	O	I	I
B5	4.2000	0.0226	0.0239	O	M	O	O
B6	4.0622	0.0218	0.0221	O	O	I	M
C1	4.1000	0.0216	0.0211	I	O	I	I
C2	3.6054	0.0201	0.0200	I	I	I	I
C3	4.6081	0.0232	0.0246	O	C	O	O
C4	4.2054	0.0217	0.0223	M	O	I	M
C5	3.6676	0.0213	0.0204	I	A	I	I
C6	3.7811	0.0212	0.0222	M	O	I	I
C7	4.3405	0.0229	0.0236	O	M	I	I
C8	4.0027	0.0213	0.0217	I	O	I	I
C9	4.0973	0.0221	0.0226	O	O	I	O
C10	3.9568	0.0220	0.0213	A	O	I	A
C11	3.9649	0.0214	0.0218	M	O	I	I
D1	3.9432	0.0225	0.0209	A	O	I	A
D2	4.4676	0.0229	0.0225	O	A	O	O
D3	4.1189	0.0223	0.0226	O	O	O	O
D4	4.6541	0.0234	0.0237	O	C	O	O
D5	4.4486	0.0225	0.0230	O	M	O	O
D6	4.1270	0.0218	0.0217	A	O	I	M
D7	4.3892	0.0222	0.0231	O	M	O	O
D8	4.2703	0.0217	0.0226	M	O	I	M
D9	4.3865	0.0226	0.0230	O	M	O	O
D10	4.2459	0.0218	0.0224	O	O	I	M
E1	4.3757	0.0226	0.0207	A	O	I	O
E2	4.1351	0.0218	0.0204	A	A	I	I
E3	3.3919	0.0197	0.0179	I	I	I	I
E4	3.5784	0.0199	0.0184	I	I	I	I
E5	3.8595	0.0211	0.0194	I	A	I	I
E6	3.8297	0.0204	0.0196	I	I	I	I
E7	3.9622	0.0214	0.0202	I	A	I	I
E8	3.9514	0.0213	0.0196	I	A	I	I

Among the identified requirement categories, the Candy model yielded the highest number of categories, whereas the traditional method resulted in the lowest (Table 10). Additionally, across the four different methodologies, the counts of attractive and must-be are minimal or absent. The scarcity of attractive requirements may align with finding that older adults exhibit greater risk aversion^72^, particularly when these services involve core interests such as financial security or health privacy. What’s more, previous research shows that an attribute will evolve from one property to another over time, conforming to the I → A → O → M life cycle trajectory. Therefore, the reason for the lack of must-be is that HBEC is in its infancy, and with the increase of time and the emergence of market competition, this category will gradually increase in the future.

**Table 10:** Comparison of the number of requirement categories obtained by different methods.

Category	Ratio model	Candy model	Normal method	BW method
O	21	21	13	18
I	15	7	33	20
M	4	7	0	5
A	6	6	0	3
C	0	5	0	0

Based on the above discussion, this study uses the results of Candy model for analysis. According to the analysis results, a total of 21 kinds of one-dimensional requirements, 7 kinds of indifferent, 7 kinds of must-be, 6 kinds of attractive and 5 kinds of critical requirements are obtained. Combined with the importance analysis, the importance rankings of different service categories are shown in Table 11**.** The priority of different categories is observed as follows: C > M > O > A > I (Table 11). Prior research has established a ranking of M > O > A > I in terms of the importance of different categories within the traditional Kano model^73^. This study not only provides intuitive support for the reliability of prior findings but also reinforces the notion that critical requirements constitute the most crucial category. Furthermore, previous studies can be leveraged to validate the methodology proposed in the current research.

**Table 11:** Importance ranking of service items by requirement category.

Category	Ranking status	Max	Min	Average
C	A6 = D4 > B1 > C3 > A5	4.6541	4.5946	4.6303
M	D5 > A4 > D7 > D9 > C7 > A1 > B5	4.4486	4.2000	4.3486
O	E1 > D8 > D10 > A8 > A10 > B2 > C4 > A2 > D6 > D3 > C1 > C9 > B6 > C8 > C11 > C10 > D1 > B3 > A9 > C6 > B4	4.3757	3.5703	4.0614
A	D2 > E2 > E7 > E8 > E5 > C5	4.4676	3.6676	4.0072
I	E6 > A3 > C2 > E4 > E3 > A7 > A11	3.8297	3.0027	3.4826

By multiplying satisfaction and dissatisfaction scores by 100 and conducting correlation analysis with importance degree, the importance shows an extremely strong positive correlation with both satisfaction (*r* = 0.924, 95% CI [0.856, 0.967], *p* < 0.001) and dissatisfaction (*r* = 0.876, 95% CI [0.790, 0.934], *p* < 0.001). Additionally, importance is highly correlated with the sum of satisfaction and dissatisfaction (*r* = 0.927, 95% CI [0.826, 0.963], *p* < 0.001). These effect sizes far exceed the 0.50 threshold for large effect sizes, indicating that importance significantly amplifies emotional responses, whether in the positive direction (satisfaction) or negative direction (dissatisfaction). The findings of this study prove that the service items that consumers feel are important have a greater probability of satisfaction and dissatisfaction, and more important service items are more likely to cause significant changes in attitude.

From an importance perspective, across various service categories, the requirement categories of diverse service items generally follow the order of C > M > O > A > I. In the contexts of smart services and security services, this ranking experiences fluctuations. Notably, intelligent reminders for going out (B2) and anti-fraud publicity campaign (D2) occupy prominent positions in terms of importance. Regarding the average importance of different service categories, security services rank highest in importance, followed by moderate importance attributed to other services, with spiritual services occupying the lowest position (Table 12). This indicates that respondents are particularly sensitive to potential losses, while spiritual entertainment and other pursuits are deemed less significant. This disparity may stem from a lack of spiritual appeal among contemporary elderly populations or the significant substitution effect played by online entertainment.

**Table 12:** Sequential distribution of importance rankings and requirement categories.

Service category	Importance ranking	Average
Life service	A6(C) > A5(C) > A4(M) > A1(M) > A8(O) > A10(O) > A2(O) > A9(O) > A3(I) > A7(I) > A11(I)	4.0292
Intelligent service	B1(C) > B2(O) > B5(M) > B6(O) > B3(O) > B4(O)	4.0860
Health service	C3(C) > C7(M) > C4(O) > C1(O) > C9(O) > C8(O) > C11(O) > C10(O) > C6(O) > C5(A) > C2(I)	4.0300
Security service	D4(C) > D2(A) > D5(M) > D7(M) > D9(M) > D8(O) > D10(O) > D6(O) > D3(O) > D1(O)	4.3051
Spiritual service	E1(O) > E2(A) > E7(A) > E8(A) > E5(A) > E6(I) > E4(I) > E3(I)	3.8855

### Differentiated requirement analysis and service design

Merely analyzing the overall requirement level may overlook the disparities in requirements among specific elderly subpopulations, potentially resulting in the concealment or underestimation of the genuine needs of particular groups. To address this issue and achieve a more precise identification of requirements, it is imperative to adopt a group-difference perspective. This approach necessitates a thorough exploration of the specific and differentiated requirements of diverse elderly care groups for HBEC services, from various viewpoints. By doing so, we can more comprehensively comprehend the nuanced differences in requirements among groups characterized by varying economic statuses, relationships with children, geographic proximities to children, and self-care abilities in the context of HBEC services. As depicted in Table 13, distinct groups exhibit notable variations in their requirement for service items, particularly evident across approximately 20 service items. These items are roughly uniformly distributed across five diverse service categories, each characterized by differentiated requirement traits.

**Table 13:** Characteristic analysis of differentiated Kano requirements.

Service content	Perspective		Income		Relationship		Distance		Self-care	
	Child	Old	Low	High	Bad	Good	Far	Near	Poor	Good
Samples	183	187	281	89	156	214	191	179	138	232
A1	M	**A**	M	M	M	M	M	**O**	M	M
A2	O	O	O	O	O	O	O	O	O	O
A3	I	I	I	**A**	I	I	I	I	I	**A**
A4	M	M	M	M	M	M	M	M	M	M
A5	C	C	C	C	C	C	C	C	C	C
A6	**M**	C	C	C	C	C	C	C	C	C
A7	I	I	I	I	I	I	I	I	I	I
A8	O	O	O	O	O	O	O	O	O	O
A9	O	**M**	O	O	O	**M**	O	**M**	**M**	O
A10	O	O	O	O	O	O	O	O	O	O
A11	I	I	I	I	I	I	I	I	I	I
B1	C	C	C	C	C	C	C	C	C	C
B2	O	O	O	**A**	O	O	O	O	O	O
B3	O	O	O	O	O	O	O	O	O	O
B4	O	O	O	O	**A**	O	O	O	O	O
B5	M	M	M	M	M	M	M	**C**	M	M
B6	O	O	O	O	O	O	O	O	O	O
C1	O	O	O	O	O	O	O	O	O	O
C2	I	I	I	I	I	I	I	I	I	I
C3	C	C	C	C	C	C	C	**M**	C	C
C4	O	O	O	O	O	O	O	O	O	O
C5	**O**	A	A	**O**	A	**O**	**O**	A	A	A
C6	O	O	O	O	O	O	O	O	O	O
C7	C	**M**	C	**M**	C	**M**	C	**M**	C	**M**
C8	O	O	O	O	O	O	O	O	O	O
C9	O	O	O	O	O	O	O	**M**	O	O
C10	O	O	O	O	O	O	O	O	O	O
C11	O	O	O	O	O	O	O	O	O	O
D1	O	O	O	O	O	O	O	O	O	O
D2	O	O	**A**	O	**C**	O	O	**A**	**A**	O
D3	O	O	O	O	**M**	O	O	O	O	O
D4	C	C	C	C	C	C	C	C	C	C
D5	**O**	M	M	M	M	**O**	M	**O**	M	**O**
D6	O	O	O	O	O	O	O	O	O	O
D7	M	M	M	M	M	M	M	**O**	M	M
D8	O	O	O	O	O	O	O	O	O	O
D9	M	M	M	**O**	**C**	M	**O**	M	M	**O**
D10	O	O	O	O	O	O	O	O	O	O
E1	O	O	O	O	O	**A**	**A**	O	O	O
E2	A	A	A	**O**	**O**	A	A	**O**	**O**	A
E3	I	I	I	I	I	I	I	I	I	I
E4	I	I	I	I	I	I	I	I	I	I
E5	A	A	A	A	A	A	A	A	A	A
E6	I	I	I	I	**A**	I	I	**A**	**A**	I
E7	A	A	A	A	A	A	A	**O**	A	A
E8	A	A	A	A	A	A	A	A	A	A

Significant values are in bold.

It can be observed that there exist disparities in requirement categories among groups with varying income levels, child relationships and so on. These variations in requirement can be explained by the principles governing requirement changes and are further influenced by group psychological characteristics. From a change perspective, according to the requirement transition pattern I → A → O(C) → M, variations in a specific requirement primarily oscillate between two similar types of requirements. This suggests that requirement changes propagate from specific groups to the entire population as a process. Regarding group psychology, factors such as income level, proximity to children, living distance, and self-care ability of different groups influence their expectations regarding various service items. Groups with differing perspectives also harbor distinct expectations due to variations in their roles and circumstances. High-income groups exhibit heightened expectations for novel services and demonstrate greater tolerance for labor-intensive offerings. Conversely, those with poor relationships with their children prioritize property security and aspire to better spiritual communication. Individuals who live in close proximity are influenced by conditions of responsibility, leading elderly individuals to prefer independent social interactions and activities. Meanwhile, those with limited self-care abilities have a heightened need for healthcare services but also display greater tolerance in other aspects.

In terms of service design, providers, policymakers, and technology developers may proceed as follows. Firstly, Eliminate or reclassify indifferent attributes as optional services. Such screening can help avoid unnecessary investment mistakes by the government, investors, or developers, thereby improving the efficiency of resource utilization. Secondly, the prioritization of service importance should adhere to the hierarchy of C > M > O > A > I. Ensuring the satisfaction of critical and must-be requirements forms the foundation of HBEC services, while addressing one-dimensional requirements serves as an effective extension, and fulfilling attractive requirements represents the pinnacle of service excellence. Prioritizing service items by importance enables better alignment of resource allocation and offers clear guidance for policy support, government oversight, process optimization, resource deployment, product design, and technology innovation. Thirdly, when considering differentiated groups, their unique requirements should be taken into account. Depending on the requirements of these diverse groups, services can be standardized according to the highest benchmarks to minimize administrative costs, or differentiated based on varying standards to enhance service satisfaction. Service offerings and their attribute classifications should be reassessed at regular intervals to keep pace with evolving needs and expectations. The government should focus on the fundamental needs of older adults, providing essential support, oversight, or assistance for the full range of differentiated services. In terms of technological development, attention should be paid to the diversity of needs, driving differentiated R&D and product design. Lastly, from the multi-stakeholder perspective, the implementation of service projects must account for factors such as differing viewpoints, shifts in responsibility conditions, the dialectical unity of conflicting requirements, and potential changes in requirement characteristics.

## Discussions

Drawing upon the development trajectory and societal context of elderly care in China, this study utilized multi-source heterogeneous data that incorporated relevant literature, online review data, and multimedia sources. Employing the GT method, it took into account the unique characteristics of the HBEC setting, advancements in intelligent technology, elderly needs, and the requirements of multiple stakeholders. Through this process, 46 distinct service items were identified and finalized. These services encompass five critical dimensions: daily living, intelligence and technology integration, health management, safety, and spiritual well-being, collectively covering nearly all major facets of HBEC. To gather empirical data, a questionnaire survey was conducted, resulting in the collection of over 400 responses. After processing, 370 valid questionnaires were retained, primarily sourced from the families of teachers and students at a university. Given the unique characteristics of the research subject influenced by Chinese traditional culture, this study refined the Kano model into a “Candy model” to more effectively mine the pertinent information embedded within the data. This enhancement involved expanding the types of attributes and bolstering the reliability and robustness of the results.

Utilizing the Candy model for data analysis, this study identified 21 one-dimensional requirements, 7 indifferent requirements, 7 must-be requirements, 6 attractive requirements, and 5 critical requirements. Among these, the critical requirements are primarily focused on indoor anti-slip and anti-fall service (A6), emergency call response system (D4), wearable emergency call device (B1), regular physical examination service (C3), and heavy lifting service (A5). These requirements exhibit high sensitivity in both the satisfaction and dissatisfaction ranges, representing the primary categories of requirements that service providers must address. Must-be requirements mainly encompass service personnel reliability audit (D5), maintenance service (A4), and service process audio and video recording system (D7). These are fundamental services that must be delivered, and their satisfaction not only impacts the specific attribute but also influences the satisfaction of other requirement categories. One-dimensional requirements primarily consist of chat and social activities (E1) and indoor property registration service(D8), which are highly regarded and relatively important service items. The attractive requirements mainly involve anti-fraud publicity campaign (D2) and psychological counseling service(E2), which have the potential to significantly enhance overall satisfaction.

Through a comparative analysis of various methodologies, it is determined that the Candy model, grounded in questionnaire survey data in this study, yields results with superior resolution and robustness. In contrast to traditional methods, the Candy model mitigates sample interference by transforming absolute values into relative ones, thereby facilitating the extraction of pertinent information. When compared to the simple ratio method, the Candy model introduces interval values as the basis for categorizing attributes, enhancing the robustness and rationality of the findings. This refinement shifts from absolute to relative metrics, extracting differentiated signals masked by homogeneous data and thereby pinpointing the true demand characteristics of genuinely needy users. Furthermore, it incorporates an additional requirement type, thereby enriching the results. This new calculation way captures service requirements to which consumers are more sensitive, enables finer-grained categorization of requirements, and thus provides more targeted guidance for service optimization and resource allocation. In comparison to the BW method, the Candy model not only identifies a novel requirement category but also tends to analyze requirement categories holistically, better capturing the commonalities within groups during differentiation analysis.

By integrating importance analysis, this study empirically validates the Candy model’s scientific robustness in HBEC service design. Specifically, the newly identified critical requirements demonstrate the highest priority, with the importance hierarchy as follows: C > M > O > A > I. Notably, the importance of one-dimensional and attractive is comparable, while significant differences exist among the other requirement categories. The ranking of traditional requirement categories aligns with previous research, thereby validating the efficacy of the Candy model. At the same time, the satisfaction and dissatisfaction characteristics exhibited by respondents are intimately linked to the perceived importance and expectation levels associated with various requirements. Specifically, when a requirement is deemed highly important, heightened expectations tend to position it closer to must-be requirements, whereas lower expectations align it more with attractive requirements. Through differential analysis, it can be seen that needs often change between two adjacent needs within a specific context. Changes in internal and external factors shape users’ expectations and their perception of importance, which in turn manifests as shifting requirement characteristics for different services. Market participants should monitor these dynamics closely and adapt service offerings and standards accordingly.

The findings of this study reflect distinct Chinese cultural characteristics and reveal how sociocultural shifts shape attitudes toward elder care. It is generally assumed that when older parents enjoy close emotional ties or live near their children, their demand for formal care services diminishes, because the children substitute for such services^74^. Surprisingly, our findings show that elders who live nearby or report better relationships still express high expectations for items such as A9 and D2, contradicting prior evidence. This divergence may stem, on the one hand, from contemporary adult children’s chronic shortages of time and energy, and on the other, from the responsibility ethic^75^: when confronted with filial obligations, older parents may feel compelled to relieve their offspring’s burden by seeking additional support without expecting reciprocation. This is a new phenomenon emerging under the combined pressures of work and shifting social norms. To account for it, we introduce the concept of “responsibility conditions”—specific conditions in which an adult child who fails to care for their parents will be judged, by themselves or others, as unfilial. Such conditions include living nearby, co-residence, or being an only child. Under these responsibility conditions, we observe parents extending understanding toward their children, manifested in their marked demand for certain support services. Responsibility conditions emerge from the time conflict between employment and filial obligations, a universal issue in modern societies. When such conditions prevail, parents’ understanding toward their children further expands the HBEC market, because it effectively liberates both parties from their ethical dilemma.

China is currently confronted with unprecedented pressures stemming from population aging, beyond formulating policy and exercising oversight, the government must also extend targeted support to specific groups to avert human tragedies or social unrest. Given the historical implementation of the family planning policy, the government bears a certain responsibility towards parents who have lost their only child, particularly those within this population who lack financial means. While economic and social disparities in elderly care services resulting from the wealth gap are generally acceptable, these low-income “shidu” (empty-nest elderly whose only child has deceased) parents may be unable to afford the costs associated with HBEC, potentially undermining the credibility of the government due to their tragic circumstances. Non-governmental social forces, such as volunteer services and charitable assistance, can serve as a buffer, yet their rights and interests are prone to infringement in the absence of government oversight. Future research endeavors can explore and analyze potential solutions to the issue of HBEC for low-income individuals, particularly low-income shidu individuals, through the investigation of time banking, multi-party supervision, and other innovative models of elderly care research.

These findings are applicable within China; internationally, cultural differences should be taken into account. Although our sample is drawn exclusively from Shandong and neighboring provinces, it is nevertheless representative of China as a whole. The country is a major educational power whose curriculum is uniform nationwide, and its economic and social development has proceeded in near-lockstep across regions. In recent years, the pervasive reach of short-video and other multimedia platforms has further accelerated cultural convergence throughout the nation. When applying this study in other regions, the research methodology employed here is broadly applicable across regions and countries, as it refines earlier approaches and demonstrates superior robustness and validity in comparative analyses. The service items were designed from the realities of China and already span most elder-care requirements; nevertheless, any region-specific requirements should be added during replication. Findings on requirement characteristics can serve as a key reference and benchmark: basic needs are likely to display similar patterns worldwide, whereas needs sensitive to context may vary across locales. Future research can innovate in methodology, subjects, and conceptual framing, such as developing more effective techniques, launching HBEC studies in new regions, adopting alternative criteria for group segmentation, focusing on specific cohorts, or conducting dynamic analyses.

## Data Availability

The brief data can be provided on request by contacting zdf_1987@163.com (Dianfeng Zhang).
